# Contamination of CT scanner surfaces with SARS-CoV-2 and infective potential after examination of invasively ventilated, non-invasively ventilated and non-ventilated patients with positive throat swabs: prospective investigation using real-time reverse-transcription PCR and viral cell culture

**DOI:** 10.1186/s13244-022-01202-x

**Published:** 2022-03-28

**Authors:** Friedemann Göhler, Victor M. Corman, Tobias Bleicker, Andrea Stroux, Marc Dewey, Torsten Diekhoff

**Affiliations:** 1 Department of Radiology, Charité - University Medicine Berlin, Charitéplatz 1, 10117 Berlin, Germany; 2 Institute of Virology, Charité - University Medicine Berlin, Charitéplatz 1, 10117 Berlin, Germany; 3 Institute of Biometry and Clinical Epidemiology, Charité - University Medicine Berlin, Charitéplatz 1, 10117 Berlin, Germany

**Keywords:** SARS-CoV-2, COVID-19, Disease transmission (Infectious), Cross infection

## Abstract

**Background:**

During the current severe acute respiratory syndrome coronavirus type 2 (SARS-CoV-2) pandemic, computed tomography (CT) has become widely used in patients with suspected or known coronavirus disease 2019 (COVID-19). This prospective observational study in 28 invasively ventilated and 18 non-invasively ventilated patients with confirmed SARS-CoV-2 contamination aims at investigating SARS-CoV-2 contamination of CT scanner surfaces and its infectiousness.

**Methods:**

Swab sampling of the CT table and gantry before and after CT examinations was performed. Additionally, the CT ventilation system air grid was wiped off after each examination. Real-time reverse-transcription polymerase chain reaction (RT-PCR) for SARS-CoV-2 RNA (ribonucleic acid) and viral cell culture were performed in the virology core lab.

**Results:**

After examination of non-invasively ventilated or non-ventilated patients, SARS-CoV-2 RNA was found in 11.1% (4/36) on patient near surfaces (CT table and gantry) and in 16.7% (3/18) on the CT air grid respectively after examination of invasively ventilated patients in 5.4% (3/56) on CT table and gantry and 7.1% (2/28) on the CT air grid. Surface contamination was more common in non-invasively ventilated or non-ventilated patients with a high viral load who were actively coughing. RT-PCR cycle threshold (Ct) was high (35.96–39.31) in all positive samples and no positive viral cell culture was found.

**Conclusion:**

Our study suggests that CT scanner surface contamination with SARS-CoV-2 is considerable and more common after examination of non-invasively ventilated or non-ventilated patients compared to invasively ventilated patients. However, no viral cell culture positivity was found, hence the infectious potential seems low.

## Key points

- Contamination of patient near CT scanner surfaces (CT table/CT gantry) with SARS-CoV-2 RNA was seen in 11.1% (4/36) of surface samples taken after examination of non-invasively ventilated and non-ventilated patients and in 5.4% (3/56) after examination of invasively ventilated patients.
- Contamination of the CT ventilation system air grid with SARS-CoV-2 was found in 16.7% (3/18) of surface samples taken after examination of non-invasively ventilated and non-ventilated patients and in 7.1% (2/28) after examination of invasively ventilated patients.
- Surface contamination was more frequent in non-invasively ventilated and non-ventilated patients with a high viral load who were coughing.
- No positive viral cell culture was found; hence the infectious potential of found virus material on CT scanner surfaces seems low.

## Background

During the current pandemic of the novel severe acute respiratory syndrome coronavirus type 2 (SARS-CoV-2), computed tomography (CT) has emerged as a valuable tool for detection of viral pneumonia in the initial diagnostic assessment of patients with coronavirus disease 2019 (COVID-19), caused by SARS-CoV-2, as well as for detection of complications in the further clinical course of these patients, who often suffer from coagulopathy.

Beside the major route of SARS-CoV-2 transmission via virus containing respiratory particles, various further infection routes are described in the literature like direct contact with an infected person, fecal–oral transmission as well as surfaces that were in direct touch with an infected person or on which virus-containing droplets have landed [2–5]. While virus-containing droplets, expired by SARS-CoV-2-infected persons, deposit quickly on surfaces close to the emission point [3, 5], virus-containing aerosols can remain airborne for many hours [4]. In view of these routes of virus transmission, proper air decontamination and surface disinfection are recommended after CT examination of SARS-CoV-2-positive patients [6, 7]. While air contamination in the examination room is addressed by ventilation systems and protection through medical face masks/respirators, surface decontamination of larger surfaces is done by specific disinfectants and often requires exposure for a couple of minutes [6]. This time factor can cause concerns when sudden emergency imaging is required, and the risk of nosocomial infection through the CT examination, caused by insufficient disinfection, is unknown.

In the existing literature, we found no study comprehensively addressing the aspect of surface contamination with SARS-CoV-2 virus material and its infective potential in the specific context of CT examinations, which are often performed with a high patient throughput, short patient stays in the examination room, and a heterogeneous patient clientele. There were only a few studies that found SARS-CoV-2 RNA on surfaces in the CT examination room while not providing information on the infective potential of the found viral material or detailed patient and examination characteristics [8, 9].

Therefore, the purpose of this study was to add more evidence of the SARS-CoV-2 transmission risk through CT examinations, investigating the presence and infective potential of SARS-CoV-2 RNA on CT scanners with specific examination and patient characteristics.

## Material and methods

For this monocentric prospective observational pilot study, we took swab samples from two CT scanners before and after scanning patients with SARS-CoV-2-positive throat swabs in a university medical center between February and May 2021. One of these CT scanners was an 80-slice machine (Canon Aquilion PRIME, Canon Medical Systems), the other a 16-slice scanner (Symbia Intevo 16, Siemens Healthcare GmbH).

Inclusion criteria for the study were patient age over 17 years and SARS-CoV-2 infection, confirmed by RT-PCR (real-time reverse-transcription polymerase chain reaction), with ongoing isolation and positive SARS-CoV-2 RT-PCR in the most recently taken throat swap. Patients were grouped into invasively ventilated and non-invasively ventilated or non-ventilated patients to segregate effects from closed ventilation systems in patients with SARS-CoV-2, which is mainly transmitted through respiratory particles [2]. The local ethics committee approved this anonymous observational study and provided a waiver for written informed consent (EA1/085/21).

### Sampling

Swab samples were taken with an industrial swab system containing 1 mL liquid Amies preservation medium in the transport vial (ESwab, Copan Italia S.p.a.). Before sampling, swabs were prewetted in physiologic saline solution (0.9% NaCl, B. Braun).

**Fig. 1:**
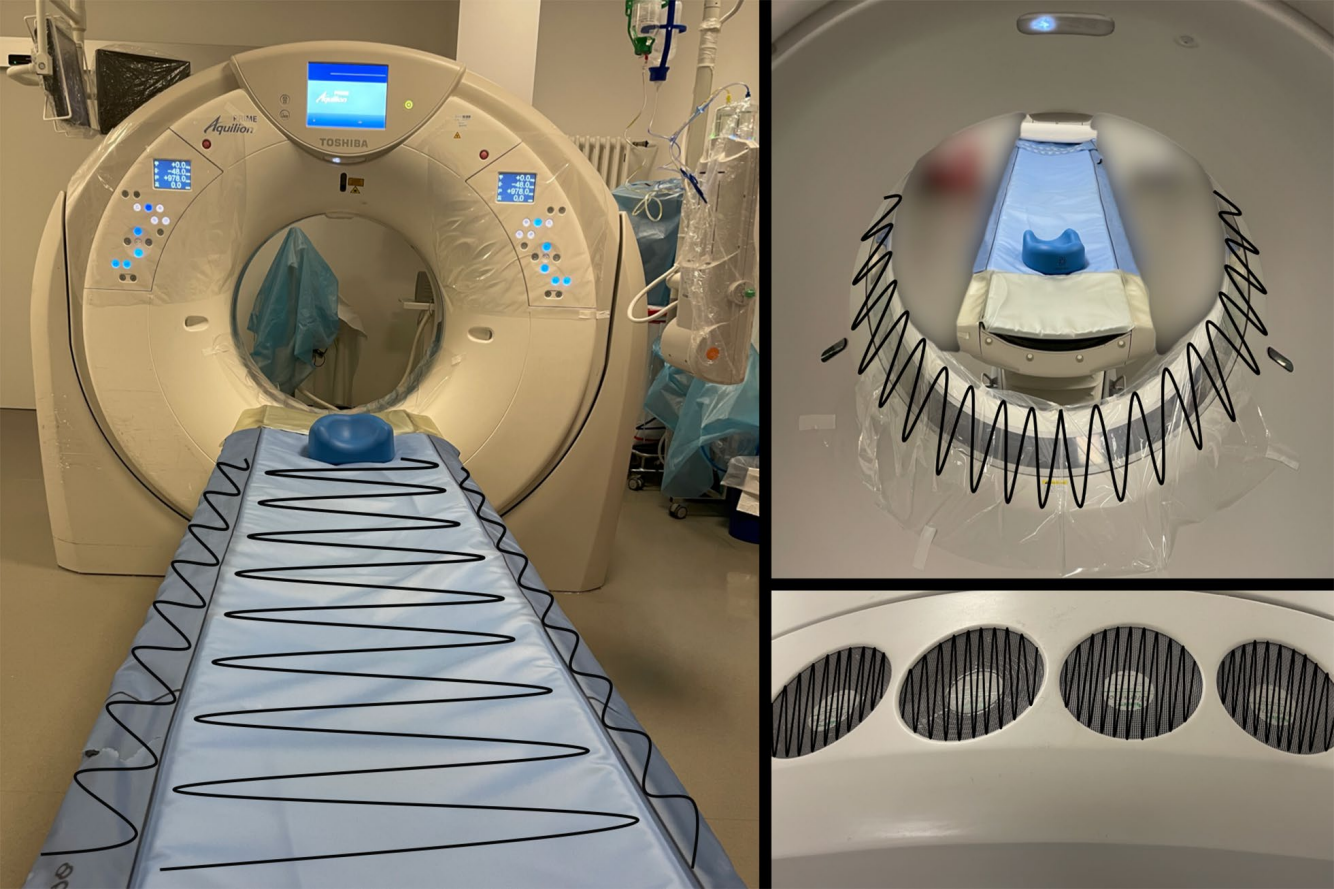
Schematic presentation of sampling procedure. Black lines: paths of swap sampling on CT table, CT gantry and CT ventilation system air grid. CT gantry was wiped off 360°

Samples were taken from the clean CT scanner table and the CT gantry (360°) before the patient entered the examination room and from the same sides, after the patient left the room, but before disinfection started. One additional sample was taken from the CT ventilation system air grid after the examination. The schematic sampling procedure for one scanner is shown in Fig. 1 and was performed in the same manner on the second CT scanner. Upon completion of swab sampling, the CT scanner and examination room surfaces were disinfected according to our institutional guidelines with 1% Incidin (Ecolab Deutschland GmbH) and 15 min exposure time. After sampling, swabs were timely transported in the preservation medium containing transport vials to the Institute for Virology in our hospital for RT-PCR and viral cell culture analysis.

### Patient data

Patient-related data were collected from our radiology information system (RIS). These data included age, sex, date of symptom onset, date of first positive RT-PCR, date of last positive RT-PCR, cycle threshold (Ct) value of the last positive RT-PCR and presence/absence of a virus mutation.

On site, the ventilation status and the type of face mask (if applicable) were noted. During the examination, a radiologist observed the CT suite through the control room window and recorded noises via the in-room microphone to register relevant events such as coughing, spreading of body fluids through disconnection of devices with direct contact to body fluids, disconnection of mechanical ventilation system or other aerosol-generating events such as intubation and resuscitation.

### Sample processing

After sampling, specimens underwent RT-PCR for testing the presence of SARS-CoV-2-specific RNA, targeting the Sarbecovirus envelope gene (E gene) and nucleocapsid protein gene (N gene) (TIB Molbiol). RNA was extracted using 200 µl and the Viral NA Small Volume Kit and the MagNA Pure 96 system (Roche) [10]. Furthermore, viral cell culture was performed to detect the cytopathic effect of the detected SARS-CoV-2 virus material as a proxy for infectiousness as described before [11].

### Statistical analysis

For this observational study we only performed basic descriptive statistics by calculation of absolute and relative frequencies for categorical variables; quantitative measurements are presented as range, mean and standard deviation for normally distributed data (age only) or as range, median, and quartiles (first (Q1) and third quartile (Q3)) for skewed values. For variables with four or less values the raw data are shown (RT-PCR Ct-values, RNA copies per mL, duration of examination and STT (symptom to test time) of SARS-CoV-2 positive samples/cases). Statistical analyses were performed with IBM SPSS Statistics Version 26.

## Results

### Patient characteristics

**Fig. 2:**
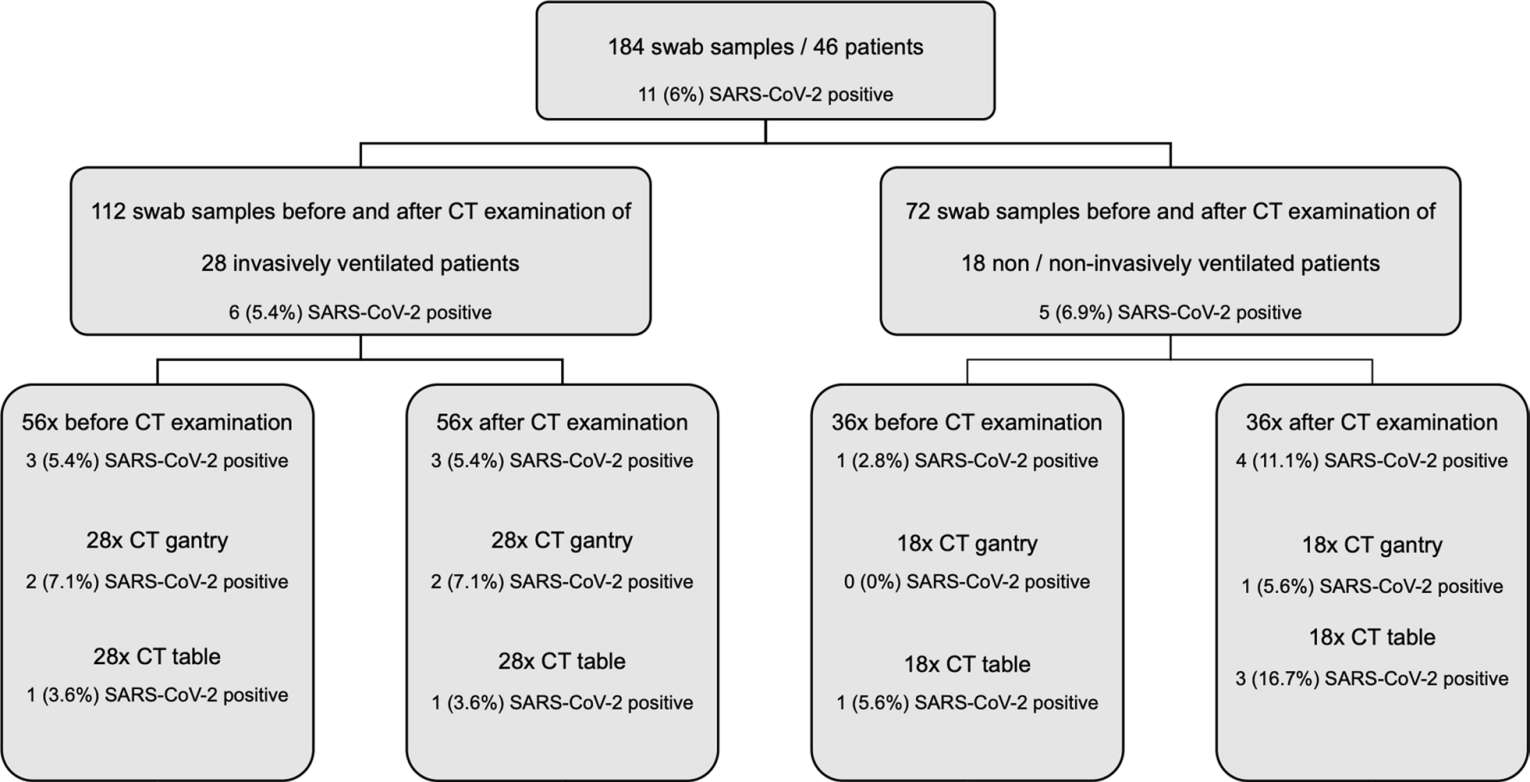
Visualization of the sampling process from patient near surfaces (CT table/CT gantry). SARS-CoV-2: severe acute respiratory syndrome coronavirus type 2; SARS-CoV-2 positive: number and percentage of positive RT-PCR results for SARS-CoV-2 RNA

We took a total of 184 swab samples from both CT table and CT gantry before and after examination of 46 patients. Additionally, we took 46 swap samples from the CT ventilation air grid after each examination. Twenty-eight patients were invasively ventilated while 18 patients had no (n = 15) or non-invasive ventilation (n = 3). A flow chart for visualization of the sampling process from CT table and CT gantry and the sample numbers is shown in Fig. 2.

In the group of non-invasively ventilated and non-ventilated patients, twelve wore an FFP-2 (filtering facepiece-2) respirator during the CT examination, three patients a mask for non-invasive ventilation (NIV), and two patients an oxygen mask while one patient tolerated no face mask during the examination. In this subset, median STT was 10 (Q1: 3.5, Q3: 12.25, range: 0–31) days, and the median time between the patient’s last positive SARS-CoV-2 RT-PCR and CT examination/test was 0.5 (Q1: 0, Q3: 4.75, range: 0–11) days. In five cases, the first positive SARS-CoV-2 RT-PCR date was set as symptom onset.

Among the invasively ventilated patients, 21 were ventilated via an endotracheal tube, and seven through a tracheal cannula. In this subset, the median duration between symptom onset and CT examination/test (STT) was 21 (Q1: 15.5, Q3: 28.75, range: 4–38) days, the median time between the patient’s last positive SARS-CoV-2 RT-PCR and CT examination/test was 8 (Q1: 3.25, Q3: 11, range: 0–22) days. In six cases with unclear symptom onset, the date of the first positive SARS-CoV-2 RT-PCR was taken as symptom onset.

Documented events during the CT examination were short disconnection of the ventilation system in three cases (10.7%) of the invasively ventilated patients and cough in 9 of 18 cases (50%) of the non-invasively ventilated/non-ventilated patients. No other unexpected aerosol or body fluid releasing events occurred. Further patient characteristics are compiled in Table 1.

**Table 1:** Patient characteristics

	No/non-invasive ventilation	Invasive ventilation
Number (sex)	18 *(female: 8/male: 10)*	28 *(female: 10/male: 18)*
Age (years; *mean* ± *SD/range)*	64.44 ± 15.72/32–88	64.43 ± 11.75/39–86
Mutation analysis		
Wild type B.1.1.7 unknown	9 (50.00%) 6 (33.33%) 3 (16.67%)	16 (57.14%) 5 (17.86%) 7 (25.00%)
STT (days)		
Median Q1/Q3 Range	10 3.5/12.25 0- 31	21 15.5/28.75 4–38
Days between most recent RT-PCR and CT		
Median Q1/Q3 Range	0.5 0/4.75 0–11	8 3.25/11 0–22
Ct value of most recent RT-PCR		
Low (< 30) High (> 30) Unknown	11 (61.11%) 4 (22.22%) 3 (16.67%)	12 (42.86%) 16 (57.14%) 0 (0%)
Duration of patient stay in the CT scanner room (min)		
Median	11	25
Q1/Q3	7/15.75	21.25/29.75
Range	6–27	10–40

Wild type: SARS-CoV-2 wild type; B1.1.7: SARS-CoV-2 variant of concern; STT: symptom to test time–time between symptom onset and CT examination; Q1: first quartile, Q3: third quartile, RT-PCR: reverse-transcription polymerase chain reaction; Ct value: RT-PCR cycle threshold

### RT-PCR/cell culture—CT table and gantry

An overview of RT-PCR positive swab samples in relation to the sample numbers is given in Fig. 2. In RT-PCR, a total of 11 of 184 samples (6%) from CT table and CT gantry were positive for SARS-CoV-2. In 92 swab samples taken before CT examination, SARS-CoV-2 RNA was found in 4 (4.4%) samples, among them 1 of 36 (2.8%) in patients without or with non-invasive ventilation and 3 of 56 (5.4%) in patients with invasive ventilation. In 92 swab samples taken after CT examination, we found SARS-CoV-2 RNA in 7 (7.6%) samples, among them 4 of 36 (11.1%) after examination of patients without or with non-invasive ventilation and 3 of 56 (5.4%) after examination of patients with invasive ventilation. Data on positive samples in invasively ventilated, non-invasively ventilated and non-ventilated patients are summarized in Table 2.

**Table 2:** Characteristics of SARS-CoV-2-positive samples from patient near surfaces (CT table/CT gantry)

Group	No	Last patient Ct-value	STT (days)	Kind of mask/ventilation	Duration of examination (min)	Events during examination	Location	Ct-value	SARS-CoV-2 RNA copies/mL	Cell culture	Virus variant
Not/non-invasive ventilated	1	N/A	1*	FFP-2	9	Cough	Air Grid	38.94	39.2	Neg	N/A
	2	< 30	4	FFP-2	8	Cough	Table	37.36	4040	Neg	WT
	3	< 30	2	NIV	18	None	Air Grid	37.08	3210	Neg	WT
	4.1	< 30	7*	NIV	15	None	Gantry	38.02	490	Neg	WT
	4.2						Table	37.24	2400	Neg	WT
	4.3						Air Grid	37.37	1890	Neg	WT
	5	< 30	7	FFP-2	12	Cough	Table	37.98	547	Neg	B1.1.7
Invasive ventilated	1	< 30	20*	Tube	21	None	Gantry	39.26	12.2	Neg	WT
	2	< 30	15	Tube	22	None	Air Grid	37.42	5730	Neg	WT
	3	> 30	35	Tube	20	None	Table	35.96	39,500	Neg	B1.1.7
	4	> 30	29*	TC	25	None	Air Grid	38.43	3060	Neg	WT
	5	< 30	26	Tube	30	None	Gantry	39.31	10.2	Neg	B1.1.7

SARS-CoV-2: severe acute respiratory syndrome coronavirus type 2; Ct value: cycle threshold value of surface sample RT-PCR (reverse-transcription polymerase chain reaction); RNA: ribonucleic acid; Cell culture: result of viral cell culture; neg. = negative; STT: symptom to test time—time between symptom onset and CT examination, n*: cases in which symptom onset was unknown and date of first positive SARS-CoV-2 RT-PCR was set as symptom onset; Most recent Ct value: RT-PCR cycle threshold of the patient’s most recent SARS-CoV-2 smear test; N/A: Not applicable; FFP-2: filtering facepiece—2; NIV: non-invasive ventilation, Tube: endotracheal tube; TC: tracheal cannula; WT: SARS-CoV-2 virus wild type; B1.1.7: SARS-CoV-2 variant of concern B.1.1.7

In the non-invasively ventilated and non-ventilated patients, four samples were positive in three different examinations (two positive samples after one examination). The RT-PCR Ct value were 37.24, 37.36, 37.98 and 38.02, the number of SARS-CoV-2 RNA copies per mL in these probes were 2400, 4070, 547 and 490. SARS-CoV-2 RNA was found in three samples from the CT table and in one gantry sample. The patients after whose examination SARS-CoV-2 RNA was found wore an FFP-2 respirator in two cases and a mask for NIV in one case. In these cases, the time of the patient stay in the CT scanner room was 8, 12 and 15 min, the STT was 4, 7 and 7 days. In two of the three patients, cough during the examination was documented.

After CT examination of the invasively ventilated patients, three samples were positive in three different examinations (one positive sample per examination). The RT-PCR Ct values of the positive samples were 35.96, 39.26 and 39.31, the SARS-CoV-2 RNA copies per mL in these probes were 39,500, 12.2 and 10.2. SARS-CoV-2 RNA was found in one swab from the CT table as well as in two gantry samples. All patients after whose examination SARS-CoV-2 RNA was found were ventilated via an endotracheal tube. In these three cases, the time of the patient stay in the CT scanner room was 20, 21 and 22 min, the STT was 15, 20 and 35 days. No events during the examination were documented.

Furthermore, in both groups (non-invasively ventilated/non-ventilated versus invasively ventilated), we found SARS-CoV-2 RNA in 4 of 92 (4.3%) samples taken before the patient entered the examination room (2 from CT gantry, 2 from CT table). In these samples, the RT-PCR Ct values were 37.44, 37.7, 37.71, 38.11 the SARS-CoV-2 RNA copies per mL in these probes were 3460, 5090, 1930 and 403. In these cases, the surface specimens taken after the CT examination were PCR-negative.

Overall, SARS-CoV-2 RNA was found on CT table and/or CT gantry after examination of 5 of the 23 patients (21.7%) with a low RT-PCR Ct value of less than 30 in a recent test versus one of 20 patients (5%) with a high Ct value (> 30).

### RT-PCR/cell culture—CT ventilation system air grid

After examination of non-ventilated/non-invasively ventilated patients SARS-Co2-2 RNA was detected in 16.7% (3/18) of the samples from the CT air grid. The RT-PCR Ct-values in these cases were 37.08, 37.37 and 38.94 with 3210, 1890 and 39.2 SARS-CoV-2 RNA copies per mL. The examination time was 18, 15 and 9 min, the STT 2, 7 and 1 days.

SARS-CoV-2 RNA was found in 7,1% (2/28) of the samples taken from the CT air grid after examination of invasively ventilated patients. The RT-PCR Ct-values in these cases were 37.42 and 38.43 with 3060 respectively 5730 SARS-CoV-2 RNA copies per mL. The examination time was 22 and 25 min, the STT 15 and 29 days.

No events during the examination of both groups were documented.

In all SARS-CoV-2-positive specimens (CT table, CT gantry, CT air grid), RT-PCR was positive for the E-gen target but negative for the N-Gen target, likely explained by the low SARS-CoV-2 RNA concentration and the slightly lesser sensitivity of this assay. Viral cell culture was negative for all specimens tested.

## Discussion

In this study, we investigated potential contamination of CT scanners after examination of SARS-CoV-2 -positive patients and found near surface viral RNA in 11.1% of non-invasively ventilated or non-ventilated patients and in 5.4% of invasively ventilated patients. The probability of positive swab samples (CT table, gantry) was higher in patients with recent RT-PCR Ct values below 30 (21.7%) than in patients with higher Ct values (> 30; 5%). Coughing seems to increase the risk of virus dissemination. Seven of twelve patients with FFP-2 masks coughed during the examination, and both positive surface samples, taken after examining patients with FFP-2 mask, were from this subgroup (2/7). As a surrogate parameter for aerosol-related contamination, probes were taken from the CT ventilation system air grid. Post-CT SARS-CoV-2 RNA was detected on the air grid in 16.7% of non-invasively ventilated or non-ventilated patients and in 7.1% of invasively ventilated patients. Among all subgroups, RT-PCR Ct values were relatively high (35.96–39.31) and SARS-CoV-2 RNA copy numbers per mL low (10.2–39,500) in all positive samples, and none of the viral cell cultures was found to be positive.

The main limitation of this pilot study is the small sample size, hampering more precise subpopulation analysis. Furthermore, no swab samples were taken from the patients on the day of CT. Instead, RT-PCR Ct values of the most recent PCR test were used which probably led to overestimation of the patients' actual viral load. Another limitation is the focus on surface samples. Further investigations beyond air grid contamination are needed to address the infection potential of virus-containing aerosols in the CT suite.

To the best of our knowledge, this is the first comprehensive analysis of SARS-CoV-2 viral load on and infectability of CT scanner following comparatively short patient stay in the examination room and high patient throughput. Matos et al. [8] analyzed contamination of internal gantry components after 180 CTs of patients with confirmed SARS-CoV-2 infection. SARS-CoV-2 RNA was only found in the inward airflow filter whereas components such as the outflow fan and fan grid were devoid of virus RNA [8]. However, they didn`t obtain swab samples from outer surfaces such as the CT gantry and table. Another study investigated surface contamination of different sites in a Wuhan university hospital during the first SARS-CoV-2 outbreak. Of 36 surface swab samples taken in the CT examination room (“CT scanning machine", according to material and methods), two were positive for SARS-CoV2 RNA (5.6%) [9]. This is in the range of surface contamination rates (CT table and gantry) obtained in our study. However, no data are provided to understand where exactly swab samples were collected from the CT scanner and about the delay between imaging of infective patients and sampling. In contrast to the present study, viral cell cultures as a marker of the infectious potential were not performed in both studies [8, 9].

According to our results of virus cell cultures, RT-PCR Ct-values and the quantity of SARS-CoV-2 copies, the infective potential of the viral material is quite low. Virus cell culture in particular, being an accepted surrogate for viral transmission and infectivity [12] by observation of cytopathic effect after inoculation of cell lines [13], was negative in all subgroups. Likewise, the RT-PCR cycle threshold (Ct) value, a semiquantitative parameter of the viral load that represents the number of RT-PCR amplification cycles needed for a target gene to exceed a threshold level [14], was relatively high in all samples (35.96–39.31). Several studies showed a strong inverse correlation between the RT-PCR Ct value and SARS-CoV-2 viral cell culture positivity [12, 15, 16]. A recent investigation found an estimated probability of 8.3% to recover infectious virus if the RT-PCR cycle threshold is higher than 35 [16]. Accordingly, the quantity of viral RNA, derived from RT-PCR, was low in all surface samples (10.2–39,500 copies/mL). For this parameter, Kampen et al. stated a probability below 5% for isolation of infectious virus when SARS-CoV-2 viral load was less than 6.63 Log10 RNA copies/mL [17]. These findings underline the low infective potential of the found virus material in our study, even in non-ventilated patients with recently low RT-PCR Ct values and STT up to seven days (n = 3, Ct: 37.24–38.02).

The further findings of the present study are in agreement with some more general studies of SARS-CoV-2 virus transmission and surface contamination. Thus, the distribution of SARS-CoV-2-positive surface samples with higher detection frequency after examination of non-invasively ventilated and non-ventilated patients with a recently low RT-PCR Ct value (< 30) reflects the fact that SARS-CoV-2 viral load and Ct values are inversely correlated [14] and that invasive ventilation is often needed in the later clinical course of patients with COVID-19, after admission to the ICU (intensive care unit), several days after symptom onset [18]. In this context, other investigators showed that, over time, the viral load in upper respiratory tract samples steadily decreased during the first ten days after symptom onset [16]. In addition, invasive ventilation systems used in COVID-19 patients are closed systems with highly effective viral filters [18]. Furthermore, the observation that more virus-positive samples were found in patients with FFP-2 respirator who were coughing during their CT examination and in patients with non–invasive ventilation is also consistent with published data. Thus, FFP-2 respirators were found to considerably reduce virus spreading from SARS-CoV-2-positive persons [19, 20], however, the effect decreases significantly if the respirator has a lousy fit [20], as can be assumed when a person is coughing. Furthermore, non-invasive ventilation is known to increase aerosol formation, and a good fit of the mask for NIV is essential to minimize the amount of aerosol [18].

As an unexpected finding, SARS-CoV-2 RNA was detected in four samples taken before patients entered the CT suite (2 from CT gantry, 2 from CT table). This may be attributable to the presence of residual virus-containing aerosols from earlier examinations that take hours to settle on surfaces [21]. Interestingly, specimens taken from the same sites after the examination were negative in all four cases. A possible explanation is that the viral load on these surfaces further decreased below the detection threshold of RT-PCR during the time of CT examination.

## Conclusions

In conclusion, the present study adds evidence that CT scanner surface contamination with SARS-CoV-2 RNA occurs after examination of ventilated, non-invasively ventilated and non-ventilated patients and plausible seems to be more frequent after CT examinations of non-ventilated or non-invasively ventilated patients, patients with a high viral load, and when patients cough during the examination. However, across all investigated subgroups, the viral load of surface contamination was low, and no viral cell culture positivity was found, so that the risk of nosocomial infection through surface contamination in the CT scanner room seems to be low in situations when disinfectant exposure time cannot be kept, e.g., in emergency imaging.

## Data Availability

The datasets used and/or analyzed during the current study are available from the corresponding author on reasonable request.

## Abbreviations

B.1.1.7: SARS-CoV-2 variant of concern B.1.1.7
COVID-19: Coronavirus disease 2019
FFP-2: Filtering facepiece-2
ICU: Intensive care unit
N/A: Not applicable
NIV: Non-invasive ventilation
RIS: Radiology information system
RNA: Ribonucleic acid
RT-PCR: Real-time reverse-transcription polymerase chain reaction
RT-PCR Ct: RT-PCR cycle threshold
Q1: First quartile
Q3: Third quartile
SARS-CoV-2: Severe acute respiratory syndrome coronavirus type 2
STT: Symptom to test time
TC: Tracheal cannula
Tube: Endotracheal tube
WT: SARS-CoV-2 virus wild type
